# Porcelain Inlays and Their Limitations

**Published:** 1904-01

**Authors:** C. E. Bentley

**Affiliations:** Chicago.


### PORCELAIN INLAYS AND THEIR LIMITATIONS.

By C. E. Bentley, D. D. S., Chicago.

Read before the Southern Wisconsin Dental Society.

In any calling of the applied sciences, the first effect of an announced new idea is to sweep before it innumerable devotees of the old and tried methods into a seething whirl of enthusiastic confusion, from which sooner or later they float back as so much flotsam and jetsam to new harbors of anchorage, to await another tidal wave which again sweeps them from their moorings, and again and again this is repeated until, by and by, they become safely anchored to those things which have been tried, and await the experience of the buffeted unfortunates before they launch their professional canoes in the eddies of misfortune and failure. This is experience.

A few within the sound of my voice remember when nothing but non-cohesive gold was used for the filling of teeth; when the high priests of that doctrine would listen to no other. Later, advocates of cohesive foil came upon the scene and the literature of the early history of dentistry records a royal battle between the champions of these two methods. But who can deny that both have left us a legacy of useful information, the employment of which has enabled us to save countless teeth for our patients.

A larger number may remember what was called the *New Departure*—a method that recognized no metal as a proper material for the saving of carious teeth, but a skilful use of the plastics was urged as the *sine qua non* of all practice. This tidal wave had a rapid rise and a more rapid subsidence, but in its wreckage many valuable bits of knowledge were gathered and they form a part of the dentist's armamentarium today, to remain for all time.

Every one remembers the cataphoric wave that swept over the dental horizon with the majesty of a meteor—the wavelet of cocaine for the extraction of teeth, etc., etc. But who has not gained by reason of these tidal waves? In each instance the residue left in the crucible of time and experience has been the few imperishable truths about which unhealthy enthusiasm and indiscriminate application built a theory which could not stand the test of time, but from which much knowledge was gained and advance made.

And thus we come to examine more critically the latest tidal wave upon whose crest so many are having a dizzy whirl today.

It will not serve my purpose to speak in detail of the technic of porcelain work, nor to compare the several methods that are under discussion. The journals have so thoroughly exploited this phase of the subject that it is taken for granted that a body of this character is entirely familiar with its technic and the methods, and discussion at this time is unnecessary.

There is, however, a tendency in the profession to run to the extreme in this matter—as I view it—and do incalculable injury to what ultimately is destined to be a rational method of saving some teeth by its employment. But its indiscriminate use, especially in its unperfected state, will surely bring it into bad repute, and prematurely discourage that lasting, healthy enthusiasm that pursues all new methods until its detail is perfected.

My excuse, then, for giving this utterance is to sound a note of warning to that class of enthusiasts who insist upon porcelain fillings in nearly all cavities, to the exclusion of all other materials. Let me be understood at the start. I am an advocate of the porcelain filling and have been using it for five years, to my satisfaction. Yet I recognize its limitations—and limitations it certainly has.

The localities and conditions that chiefly indicate porcelain fillings are:

First—Labial cavities in the six anterior teeth, and buccal cavities in bicuspids.

Second—The approximal cavities in the six anterior teeth when the cavity does not extend to the incisal edge.

Third—The six anterior teeth of children, in which the tendency to decay is marked.

Fourth—In the six anterior teeth of adults having diseased peridental membranes.

The ideal position for an inlay is upon the labial aspect of any of the six anterior teeth, and the buccal aspect of bicuspids, by reason of the fact that it is removed from the stress of mastication. It does not involve the use of many shades. We are not bothered with the "shadow problem," and the cement seems to receive the maximum of protection.

Approximal cavities in the six anterior teeth not involving the incisal edge are included for æsthetic reasons. If the incisal edge be involved, I consider it a dangerous experiment to solely depend upon the cement to overcome the leverage natural to a porcelain filling in this position, and in this connection I desire to emphasize the point that the retentionof inlays involving a part or the whole of the incisal edge is enhanced by a mechanical attachment in conjunction with cement attachment.

One of the most perplexing problems that confronts the modern dentist today is the employment of the best method for the preservation of this class of children's teeth in which caries is riotously rampant during the "susceptible period." The best practice now recognized is the extension of the margins of those cavities to the territory which is called "immune zones," and extension of the cavity to zones of immunity at which initial caries seldom if ever begins, provided normal enamel covers that part. In the employment of gold in such cases, while the tooth may be preserved for æsthetic heasons, the larger display of metal is objectionable and the tax upon the patient is still more so.

For these reasons porcelain inlays are to be preferred to gold fillings in the six anterior teeth. Whatever may be said for or against porcelain inlays, we know by this time that decay *d es not* recur around its margins. The inlay may drop out, the cement may dissolve, but decay does not recur. Therefore, it is justifiable to recommend that porcelain inlays should be employed in this class of cases, thereby saving extensive cutting and the exposure of large surfaces of metal in the anterior part of the mouth. 'Twere better to set and reset an inlay in the class of cases described until a condition of lessened susceptibility is arrived at, than to fill the mouth with gold fillings according to the laws governing extension for prevention with its attendant tendency to recurrence of decay about gold fillings, whether placed according to said laws or not, during this period of susceptibility.

In the teeth of those advanced in age, whose peridental membrances are diseased so that the malleting, incident to the placing of a gold filling would produce an irritated condition, more or less inimical to the already impaired membrane, porcelain fillings should be placed in the six anterior teeth.

In the compound cavities in bicuspids and molars, such as mesio-occlusal and disto-occlusal, I believe the gold inlay to be of far greater service than the porcelain inlay in these localities for the following reasons:

First, The anchorage.

Second, The strength of the margin.

(a) In approximal occlusal cavities the occlusal step method may be employed with advantage to the retention of the gold inlay.

(b) In gold inlays the margins are freely beveled and the edges of the gold carried over them; thus frail enamed walls may be ground down and the gold built over them, thereby adding strength to an otherwise frail wall, with no danger of crushing the material overlapping it. With a porcelain inlay in this locality the vulnerable parts would be the remaining frail wall, and, in case it were ground down, the thin edge of porcelain, which would not withstand the stress of mastication. Gold inlays in these localities are more and more being employed and their rationale becoming more and more accepted.

According to the foregoing, you will recognize the limitations of the porcelain inlay, its employment being confined to certain cavities in the ten anterior teeth for the reasons cited above.

Another reason for a plea for discrimination in its use, aside from the causes mentioned, is the lack of our knowledge of the behavior of cement in any given case. The only means of retention of an inlay is cement. In some they dissolve rapidly; in others, less so. The space occupied by cement necessary to the retention of an inlay is, indeed, small, but there is generally a washing out about the margins of the inlay shortly after it has been set that may be traced with an explorer about the entire periphery of the inlay. Dissolution of the cement seems to cease after a certain point is reached, and caries does not attack the margin. What causes this apparent cessation of cement dissolution? We shall need to await the investigations of the chemist.

Again, the same cement will act differently under different conditions. There is a difference that should be reckoned with between a freshly made cement and one that has the stamp of age upon it. There is a difference in the behavior of cements in mouths whose fluids are neutral in reaction and those that are slightly acid.

Is it not better to proceed cautiously and wait for a longer perspective before we adopt too ardently this new idea? Porcelain inlays have come to stay and will have their place among the other agencies that are at our disposal for the service demanded of us by our patients; but they should not put to riotous confusion the friends that have stood the test of time.

"If thou hast a friend and his adoption tried, grapple him to thy soul with hooks of steel," said the immortal bard. And thus I say to you about our late friends whom the porcelain enthusiasts are trying to relegate to innocuous desuetude.

Again, the young man just crossing the threshold of our profesion comes with high hopes and eager hands to try the things so loudly proclaimed from our leaders in any particular line. Too often his enthusiasm leads him to accept without question the sweeping claims of the enthusiast, and failure, accompanied with its disastrous results, is the consequence. We should be careful of our utterances in the presence of young men, and particularly is this so with this new tidal wave which is sweeping over the country. "Better stand the ills we have than fly to others we know not of" until the art is in a more perfected condition. The essential weak point of the porcelain inlay, granting that all other steps have been perfected, is its means of attachment. Until we have concentrated our best thought upon this point and evolved a method of attachment that is less liable to the ever changing conditions of the mouth, 'twere better to temper our enthusiasm with moderation, and frankly take our patients into our confidence as to the uncertainty that surrounds this beautiful work that has made for itself a place in dentistry, but still let us retain a bowing acquaintance with the old friends that have served us so well.

